# Current clinical findings of acute neurological syndromes after SARS‐CoV‐2 infection

**DOI:** 10.1002/mco2.508

**Published:** 2024-03-09

**Authors:** Minjin Wang, Jierui Wang, Yan Ren, Lu Lu, Weixi Xiong, Lifeng Li, Songtao Xu, Meng Tang, Yushang Yuan, Yi Xie, Weimin Li, Lei Chen, Dong Zhou, Binwu Ying, Jinmei Li

**Affiliations:** ^1^ Department of Neurology West China Hospital of Sichuan University Chengdu Sichuan China; ^2^ Department of Laboratory Medicine West China Hospital of Sichuan University Chengdu Sichuan China; ^3^ Institute of Brain Science and Brain‐inspired Technology West China Hospital of Sichuan University Chengdu Sichuan China; ^4^ Genskey Medical biotechnology Company Limited Beijing China; ^5^ National Institute for Viral Disease Control and Prevention Chinese Center for Disease Control and Prevention Beijing China; ^6^ Department of Respiratory and Critical Care Medicine West China Hospital Sichuan University Chengdu Sichuan China

**Keywords:** central nerve injury, Neuro‐COVID, neurological syndromes, neurotropic invasion, SARS‐CoV‐2

## Abstract

Neuro‐COVID, a condition marked by persistent symptoms post‐COVID‐19 infection, notably affects various organs, with a particular focus on the central nervous system (CNS). Despite scant evidence of SARS‐CoV‐2 invasion in the CNS, the increasing incidence of Neuro‐COVID cases indicates the onset of acute neurological symptoms early in infection. The Omicron variant, distinguished by heightened neurotropism, penetrates the CNS via the olfactory bulb. This direct invasion induces inflammation and neuronal damage, emphasizing the need for vigilance regarding potential neurological complications. Our multicenter study represents a groundbreaking revelation, documenting the definite presence of SARS‐CoV‐2 in the cerebrospinal fluid (CSF) of a significant proportion of Neuro‐COVID patients. Furthermore, notable differences emerged between RNA‐CSF‐positive and negative patients, encompassing aspects such as blood–brain barrier integrity, extent of neuronal damage, and the activation status of inflammation. Despite inherent limitations, this research provides pivotal insights into the intricate interplay between SARS‐CoV‐2 and the CNS, underscoring the necessity for ongoing research to fully comprehend the virus's enduring effects on the CNS. The findings underscore the urgency of continuous investigation Neuro‐COVID to unravel the complexities of this relationship, and pivotal in addressing the long‐term consequences of COVID‐19 on neurological health.

## Graphical abstract

Phylogeny and genomic features of 10 SARS‐CoV‐2 isolates. Notably, nine cerebrospinal fluid (CSF) samples and one matched nasopharyngeal swab (NPS) sample displayed substantial coverage of the entire SARS‐CoV‐2 genome, meeting the criteria of 10×≥90% and 100×≥60% against the original Wuhan SARS‐CoV‐2 genome. The phylogenetic tree, crafted using RAxML and visualized with iTOL, highlights consensus genomes from each analyzed sample. Sample types are denoted by the yellow square, and distinct Pango lineages, identified by Nextclade, are represented by the blue square. For clarity, three bubble plots showcase sequencing coverage (1×, 10×, and 100×) for each sample in comparison with the first Wuhan SARS‐CoV‐2 genome. Furthermore, the figure illustrates mapping and semi‐log depth of coverage of sequencing reads for each sample against the initial Wuhan SARS‐CoV‐2 genome, providing a comprehensive depiction of the genomic attributes of the examined isolates.

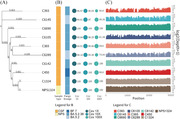

## 1 INTRODUCTION

A range of persistent symptoms can remain after infection of acute mild or severe COVID‐19, which can affect multiple organs including the central nervous system (CNS), usually collectively termed “Neuro‐COVID.”^1^, ^2^ Several signs and symptoms have been reported in Neuro‐COVID, ranging from relatively mild symptoms (such as anosmia, ageusia, headache, dizziness, etc.) to severe complications, such as seizures, encephalitis, ischemic stroke, and intracerebral hemorrhage.^3^, ^4^, ^5^ In the past, Neuro‐COVID was a relatively rare complication, with a reported incidence of 3.5% for new‐onset severe neurological events in 2020.^6^ However, since December 2022, China has experienced widespread COVID‐19 infections, leading to a rapid surge in cases, particularly among individuals who had not been previously exposed to any SARS‐CoV‐2 strains and had received non‐mRNA COVID‐19 vaccines.^7^, ^8^ Indeed, our earlier research has revealed a significant increase in the number of Neuro‐COVID patients during this period.^9^

Previous studies have supposed that, akin to viral encephalitis (VE) resulting from herpes simplex virus invasion of the CNS, the hyperimmune response and cytokine release syndrome (CRS) are likely to play pivotal roles as the primary mechanisms driving Neuro‐COVID.^10^, ^11^, ^12^, ^13^, ^14^, ^15^ Nevertheless, these studies observed that there was no evidence of widespread CNS invasion by the virus in the cerebrospinal fluid (CSF) of these patients.^10^, ^16^ Increasing instances of Neuro‐COVID cases have revealed that some patients may manifest acute neurological syndrome in the early stages of SARS‐CoV‐2 infection, even in the absence of definitive signs of hyperactivated inflammatory responses.^17^, ^18^, ^19^

Recent developments indicate a shift in the situation. Emerging SARS‐CoV‐2 variants, particularly Omicron and its successors, demonstrate altered virological characteristics, notably an increased inclination for neurotropism and neurotoxicity compared with earlier strains.^20^, ^21^ Omicron and its successor variants can infiltrate the CNS through the olfactory bulb, they not only induce inflammation in neurons, microglial cells, and astrocytes but can also directly damage neuronal cells.^22^, ^23^, ^24^, ^25^, ^26^ Indeed, the direct infection of neuronal cells has been highly recognized as a significant factor contributing to neurological complications associated with COVID‐19.^27^, ^28^ Over time, an increasing number of novel variants, such as XBB and JN.1, continue to emerge, leading to a growing interconnectedness between the virus and human existence.^29^, ^30^ Therefore, it is crucial to be vigilant about the potential neurological syndromes that may arise from the direct invasion of the CNS by SARS‐CoV‐2.^3^, ^9^, ^31^ Enhanced attention should be given to the possible neurological complications associated with SARS‐CoV‐2, given the growing evidence of its ability to cause inflammation and damage to the CNS.^32^, ^33^, ^34^

In this study, we included a group of COVID‐19 patients and described the important neurological features found in CNS after post‐SARS‐CoV‐2 invasion.

## 2 RESULTS

### 2.1 Baseline characteristics of participants

**FIGURE 1:**
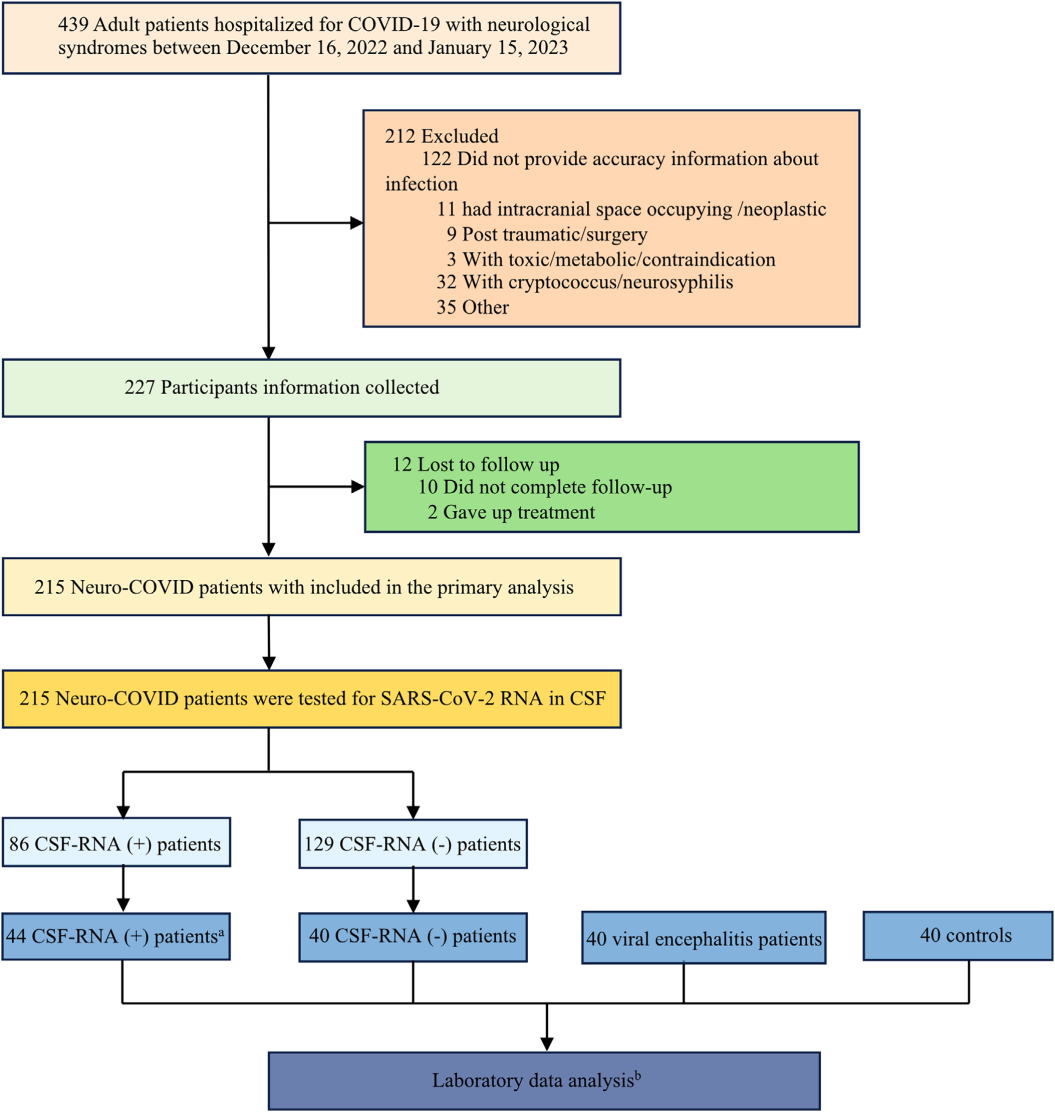
A flowchart of the study subjects. ^a^In order to ensure the integrity of WGS analysis outcomes, we exclusively opt for samples that exhibit positive results through RT‐PCR detection with a Ct < 25 for WGS analysis. ^b^Including CSF cytokines levels, total protein levels, oligoclonal bands, serum levels of neurofilament light protein and glial fibrillary acidic protein.

A total of 439 patients who had undergone diagnostic lumbar puncture were assessed, and 215 patients (median [interquartile range; IQR] age, 47 [27–58] years; 102 male [47.44%] and 113 female [52.56%]) were included in the study based on “Neuro‐COVID” criteria^1^, ^2^, ^3^ from three different medical centers in Sichuan province, China: in West China Hospital of Sichuan University (*n* = 151), in Chengdu Shang Jin Nan Fu Hospital (*n* = 34), and West China Tianfu Hospital (*n* = 20) (Figure 1). CSF samples of all patients were tested for SARS‐CoV‐2 RNA (RNA^CSF^), 86 patients exhibited detectable viral RNA (RNA^CSF^‐positive group) and no viral RNA was detected in 129 patients (RNA^CSF^‐negative group). The median cycle threshold (Ct) value for them was 31.42 (range, 27.6–35.5). There were no statistically significant differences between the two groups in clinical features, which included age, gender, baseline modified Rankin Scale (mRS), and form of onset.

### 2.2 Clinical disparities in neurological presentations

**FIGURE 2:**
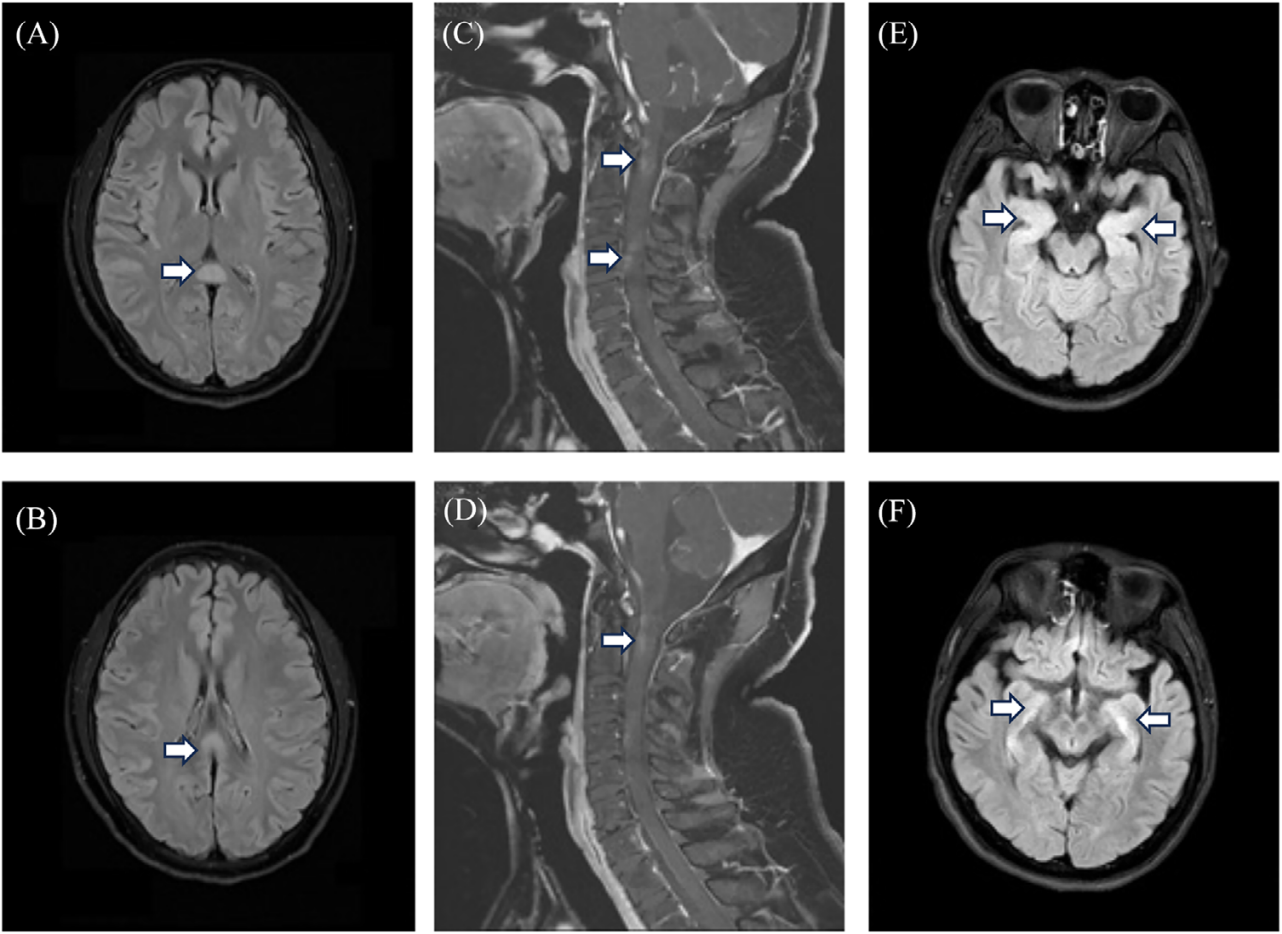
Images of brain MRI scans of Neuro‐COVID patients. (A and B) A 20‐year‐old woman presented with gradually worsening delirium after an upper respiratory tract infection. MR imaging of the brain demonstrated prominent T2‐FLAIR abnormalities in the splenium of the corpus callosum (Arrowheads). (C and D) A 55‐year‐old man with recurrent acute myelitis presented with progressively worsening numbness and weakness in the extremities. Contrast‐enhanced MRI of the cervical and thoracic spine exhibited abnormal enhancement of the cervical spinal cord and enhancement of the cervicothoracic meninges (Arrowheads). (E and F) A 19‐year‐old man presented with psychosis and behavior disorder. MRI of the brain prominent T2‐FLAIR abnormalities in the bilateral hippocampus (Arrowheads).

When analyzing the clinical differences between those with and without SARS‐CoV‐2 in their CSF, we found several significances (Table 1). First, we found that the time from confirmed COVID‐19 to the onset of any neurological syndromes in RNA^CSF^‐positive group was significantly shorter (median [IQR], 5 [2–7] vs. 7 [3–10] days; *p *= 0.048). The most frequent neurological symptoms in the RNA^CSF^‐positive group were psychosis (*n* = 53, 61.63%), disturbance of consciousness (*n* = 27, 31.4%), and seizures (*n* = 26, 30.23%). In the RNA^CSF^‐negative group, disturbance of consciousness (*n* = 36, 27.91%), psychosis (*n* = 34, 26.36%), and headache (*n* = 29, 22.48%) were the most frequently reported symptoms. Acute encephalopathy presentations of the seizures and psychosis were found to be more prevalent in RNA^CSF^‐positive group patients (30.23 vs. 7.75%; 61.63 vs. 26.36%, respectively, *p *< 0.001). The RNA^CSF^‐negative group exhibited a high incidence of the cerebellum and spinal cord injury conditions, such as limb weakness, disturbance of sensation, dizziness, and ataxia (5.81 vs. 21.71%; 1.16 vs. 15.5%; 1.16 vs. 10.85%; 0 vs. 6.2%, respectively, *p *< 0.05). A total of 87 among 215 patients (40.47%; RNA^CSF^‐positive group, *n* = 32; and RNA^CSF^‐negative group, *n* = 55) showed a past medical history in our study. In contrast, the RNA^CSF^‐negative group reported a higher recurrence probability of preexisting neurological disorders (*p *< 0.05), with eight of 86 patients (9.3%) in the RNA^CSF^‐positive group and 25 of 129 patients (19.38%) RNA^CSF^‐negative group. However, the frequencies of other associated symptoms, abnormalities in magnetic resonance imaging (MRI) examinations, treatment modalities, hospitalization time, and neurological short‐time functional outcomes were not significantly different between the two examined groups. Figure 2 demonstrates images of brain MRI scans of Neuro‐COVID patients.

**TABLE 1:** Clinical and demographic variables of Neuro‐COVID patients.

Characteristic	RNAC^SF^ positive *n* = 86	RNAC^SF^ negative *n* = 129	*p* Value
Demographic characteristics			
Age, median (IQR), years	46 (26, 59)	47 (28, 57)	0.894^a^
Sex, *n* (%), male	40 (46.51)	62 (48.44)	0.824^b^
Baseline mRS, median (IQR)	3 (3, 4)	3 (3, 4)	0.245^a^
Acute, *n* (%)	68 (79.07)	89 (68.99)	0.102^a^
Time from confirmed COVID‐19 to the onset, days, median (IQR)	5 (2, 7)	7 (3, 10)	**0.048** ^a^
WHO moderate severity, *n* (%)	2 (2.33)	2 (1.55)	0.918^b^
Neurologic symptoms, *n* (%)			
Headache	18 (20.93)	29 (22.48)	0.788^b^
Dizziness	1 (1.16)	14 (10.85)	**0.014** ^b^
Seizures	26 (30.23)	10 (7.75)	**<0.001** ^b^
Psychosis	53 (61.63)	34 (26.36)	**<0.001** ^b^
Dystaxia	0 (0)	8 (6.20)	**0.047** ^b^
Limb weakness	5 (5.81)	28 (21.71)	**0.002** ^b^
Disturbance of sensation	1 (1.16)	20 (15.50)	**0.002** ^b^
Blurred vision	6 (6.98)	10 (7.75)	0.832^b^
Cognitive disorder	10 (11.63)	18 (13.95)	0.619^b^
Disturbance of consciousness	27 (31.40)	36 (27.91)	0.582^b^
Dyskinesias and movement disorders	4 (4.65)	6 (4.65)	0.741^b^
Sleep disorders	9 (10.47)	9 (6.98)	0.366^b^
Autonomic nervous system	1 (1.16)	8 (6.20)	**0.144** ^b^
Past medical history, *n* (%)			
Preexisting neurological disorders	8 (9.30)	25 (19.38)	**0.046** ^b^
Diabetes	3 (3.49)	11 (8.53)	0.236 ^b^
Hypertension	14 (16.28)	11 (8.53)	0.082^b^
Cardiovascular disease	5 (5.81)	2 (1.55)	0.209^b^
Lung disease	1 (1.16)	1 (0.78)	0.663^b^
Kidney diseases	2 (2.33)	1 (0.78)	0.722^b^
Digestive system disease	2 (2.33)	4 (3.10)	0.932^b^
Tumor	3 (3.49)	9 (6.98)	0.275^b^
Auxiliary examinations			
Brain MRI, abnormality, *n* (%)	47/74 (63.51)	72/114 (63.16)	0.961^b^
Lung CT/chest X‐ray, abnormality, *n* (%)	84/85 (98.82)	125/127 (98.43)	0.724^b^
Treatment modalities, *n* (%)			
Antibiotic treatment	43/82 (52.44)	33/106 (31.13)	**0.003** ^b^
Antiviral treatment	67/82 (81.71)	78/106 (73.58)	0.189 ^b^
Immunotherapies			
IVIg alone	19/82 (23.17)	19/106 (17.92)	0.374^b^
IVMP alone	15/82 (18.29)	28/106 (26.42)	0.189^b^
IVIg combined with IVMP	11/82 (13.41)	10/106 (9.43)	0.390^b^
Mechanical ventilation	2 (2.33)	5 (3.88)	0.814^b^
Hospital outcomes			
Length of hospital stay, *d*, mean ± SD	11 (9, 14)	10 (8, 14)	0.1788^a^
Recovery, *n* (%)	76 (88.37)	119 (92.25)	0.918^c^
Poor response to treatment, *n* (%)	9 (10.47)	9 (6.98)	
Death, *n* (%)	1 (1.16)	1 (0.78)	

Bold entries indicate *p* < 0.05.

Abbreviations: CT, computerized tomography; IVIg, intravenous immunoglobulin; IVMP, intravenous high dose methylprednisolone.; MRI, magnetic resonance imaging; mRS, modified Rankin Scale; WHO, World Health Organization.

^a^ Mann–Whitney *U* test.

^b^ Pearson's *χ* ^2^ test. Multiple comparisons were multiplied by the number of comparisons to calculate corrected *p* (port) values (Bonferroni–Dunn correction).

^c^ Wilcoxon–Mann–Whitney test with post hoc analysis.

### 2.3 CSF analysis reveals SARS‐CoV‐2 invasion in CNS

**FIGURE 3:**
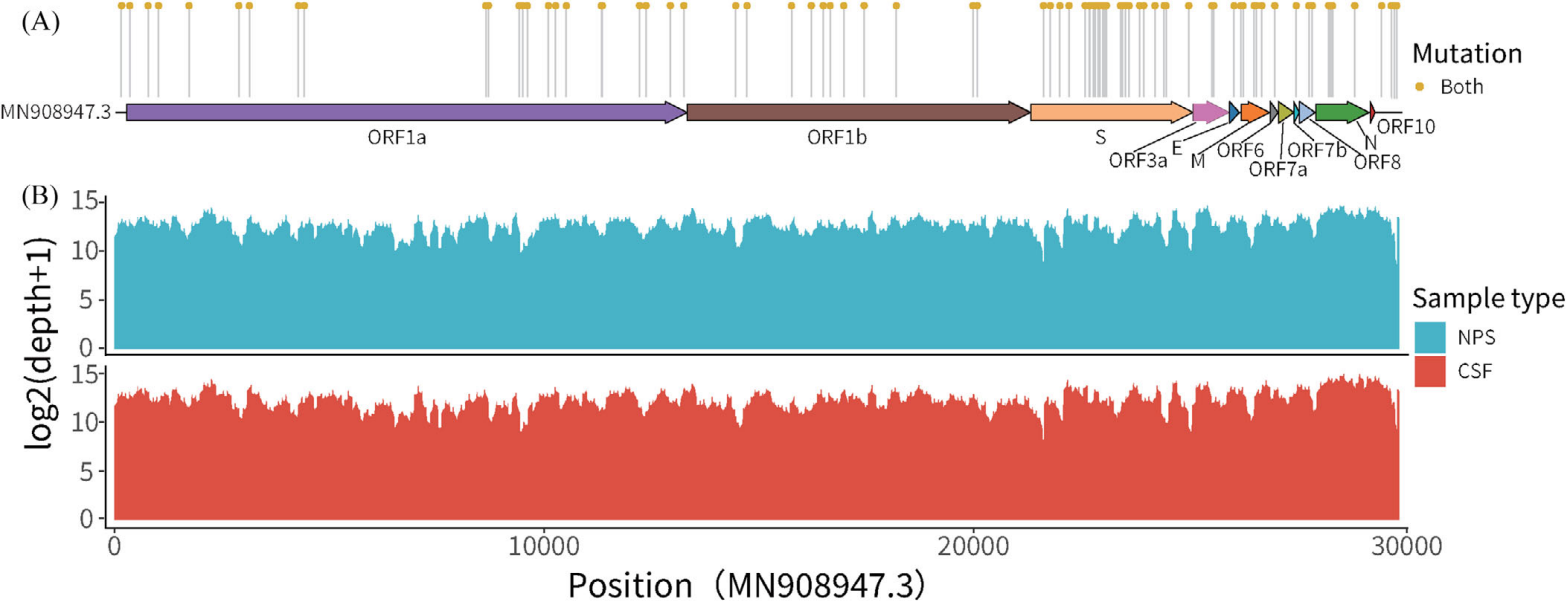
Genome statistics of SARS‐CoV‐2 isolates. Schematic representation of the genome organization and genetic mutations in the genome of SARS‐CoV‐2. Yellow dots indicate mutations found in both CSF and NPS samples. Mapping and semi‐log depth of coverage of sequencing reads in CSF and NPS samples against the first Wuhan SARS‐CoV‐2 genome.

The detection of intact viral particles in CSF represents the most reliable evidence of SARS‐CoV‐2 infiltrating the CNS.^20^, ^27^, ^28^ Hence, we selected 44 CSF samples (the RT‐PCR test results Ct < 25) and 12 matched NPS (nasopharyngeal swab) samples from RNA^CSF^‐positive patients with high‐quality RNA for virus whole viral sequencing (whole‐genome sequencing [WGS]) detection. Among them, 17 CSF samples exhibited high coverage of the complete SARS‐CoV‐2 genome (including Omicron BA.5.2 and BF.7 variants), revealing that they were not the viral genome fragments residuum (Table S1). One of the patients obtained an extremely high‐quality of the complete SARS‐CoV‐2 genome in both CSF and the matched NPS samples. Moreover, mutation analysis performed in these two samples showed a highly consistent mutation landscape (Figure 3). Evolutionary analysis showed that the CSF genomes were highly homologous to the matched NPS genomes, thus implying that the virus intracranial invasion may be closely connected with their respiratory infection ability rather than exogenous interferential (Figure S1).

The specific antigens and antibodies of the SARS‐CoV‐2 found in CSF also serve as crucial supporting evidence for its invasion of the CNS.^16^, ^18^ A total of 44 RNA^CSF^‐positive patients and 40 matched for gender, age, and mRs score RNA^CSF^‐negative patients were tested for SARS‐CoV‐2 antibodies and antigens in this part. Separate control groups were included. At the time of the SARS‐CoV‐2 antigen and antibody, prospective control samples were unavailable. Consequently, we used archived (−80°C) CSF and serum samples from age‐matched COVID−19‐negative patient controls who had undergone diagnostic lumbar puncture because of initial suspicion of CNS infection, with CSF analyses indicating no sign of ongoing CNS disease. The SARS‐CoV‐2 N‐antigen was detectable in CSF in 23 of 44 patients in the RNA^CSF^‐positive group (52.27%) (four of 23 controls). The measured CSF N‐Ag concentrations in the RNA^CSF^‐positive group were significantly higher than RNA^CSF^‐negative group. Frequencies of IgM, IgG, and neutralizing antibodies were significantly greater in RNA^CSF^‐positive group than they were in RNA^CSF^‐negative group (*p* < 0.001, respectively) (Table 2).

**TABLE 2:** SARS‐CoV‐2 related parameters and biological characteristic of Neuro‐COVID patients.

Characteristic	RNA^CSF^ positive *n* = 44	RNA^CSF^ negative *n* = 40	*p* Value
SARS‐CoV‐antigen (*Quantitative*)			
CSF N‐Ag positive, No./No.	23/44 (52.27)	4/23 (17.39)	**0.012** ^a^
N‐Ag CSF, median (range), fg/mL	6.84 (0, 40.02)	0 (0, 15.57)	**<0.0001** ^b^
CSF S‐Ag positive, No./No.	5/44 (11.36)	1/32 (3.12)	0.377^a^
S‐Ag CSF, median (range), fg/mL	0 (0, 11.29)	0 (0, 1.57)	0.104^b^
SARS‐CoV‐antibody (*Qualitative*)			
CSF IgM	24/44 (54.54)	1/32 (3.12)	**<0.0001** ^a^
CSF IgG	35/44 (79.54)	4/32 (12.50)	**<0.0001** ^a^
Neutralizing antibody	21/44 (47.72)	3/32 (9.37)	**<0.0001** ^a^
CSF detection, abnormality			
Leukocyte counts, median (IQR) (×10^6^/L)	9 (3.5, 14)	18 (9, 203)	**0.014** ^b^
Protein, median (IQR) (g/L)	0.37 (0.27, 0.61)	0.46 (0.29, 1.22)	**0.012** ^b^
OCB, positive, *n* (%)	7 (8.14)	26 (20.16)	**0.027** ^a^
CSF cytokines			
IL‐1β, median (IQR) (pg/mL)	1.53 (0.96, 2.47)	1.34 (0.95, 2.12)	0.563^b^
IL‐2, median (IQR) (pg/mL)	0.41 (0.26, 0.93)	0.51 (0.36, 0.95)	0.351^b^
IL‐4, median (IQR) (pg/mL)	0.72 (0.43, 0.93)	0.78 (0.522, 1.29)	0.349^b^
IL‐5, median (IQR) (pg/mL)	0.23 (0.13, 0.45)	0.27 (0.12, 0.49)	0.857^b^
IL‐6, median (IQR) (pg/mL)	11.57 (5.89, 15.8)	13.94 (7.89, 30.89)	0.174^b^
IL‐8, median (IQR) (pg/mL)	20.04 (8.52, 38.93)	18.77 (8.98, 42.9)	0.97^b^
IL‐10, median (IQR) (pg/mL)	1.79 (1.52, 3.62)	1.68 (1.04, 2.48)	0.265^b^
IL‐12p70, median (IQR) (pg/mL)	2.03 (1.49, 3.16)	2.3 (1.76, 3.16)	0.422^b^
IL‐17A, median (IQR) (pg/mL)	11 (6.99, 15.29)	12.51 (9.73, 20.09)	0.114^b^
TNF‐a, median (IQR) (pg/mL)	0.87 (0.62, 1.21)	1.44 (0.87, 1.64)	**0.0003** ^b^
IFN‐a, median (IQR) (pg/mL)	1.32 (0.59, 2.16)	0.82 (0.55, 2.03)	0.3^b^
IFN‐γ, median (IQR) (pg/mL)	0.64 (0.44, 1.22)	0.84 (0.45, 1.56)	0.44^b^
Plasma NfL, median (IQR) (pg/mL)	114.9 (48.59, 422.7)	17.94 (10.48, 94.12)	**<0.0001** ^b^
Plasma GFAP, median (IQR) (pg/mL)	98.05 (50.91, 220.8)	133.1 (72.77, 236.6)	0.136^b^

Bold entries indicate *p* < 0.05.

Abbreviations: CSF, cerebrospinal fluid; GFAP, glial fibrillary acidic protein.; IFN, interferon; IgG, immunoglobulin G; IgM, immunoglobulin M; IL, interleukin; NfL, neurofilament light chain; OCBs, oligoclonal bands; TNF, tumor necrosis factor.

^a^ Pearson's *χ*2 test. Multiple comparisons were multiplied by the number of comparisons to calculate corrected *p* (port) values (Bonferroni–Dunn correction).

^b^ Mann–Whitney *U* test.

To exclude infections or interferences by other pathogens appearing in atypical ways, we conducted further metagenomic next‐generation sequencing (mNGS) analysis on all CSF samples from RNA^CSF^‐positive and RNA^CSF^‐negative patients. After eliminating custom, background, and interference sequence reads, none of the samples tested positive for any pathogenic or potentially pathogenic agents. Therefore, this allows for the maximal exclusion of CNS symptoms caused by other infectious factors.

### 2.4 Key findings in the CSF of Neuro‐COVID patients

We included a comparable number of previously diagnosed VEs patients and a control group for comparative analysis within the study cohort. To assess the characteristics of neurologic damage and the integrity of the blood–brain barrier (BBB) in Neuro‐COVID patients, we conducted analyses of their CSF total protein levels, oligoclonal bands (OCBs), serum levels of neurofilament light protein (NfL), and glial fibrillary acidic protein (GFAP).

**FIGURE 4:**
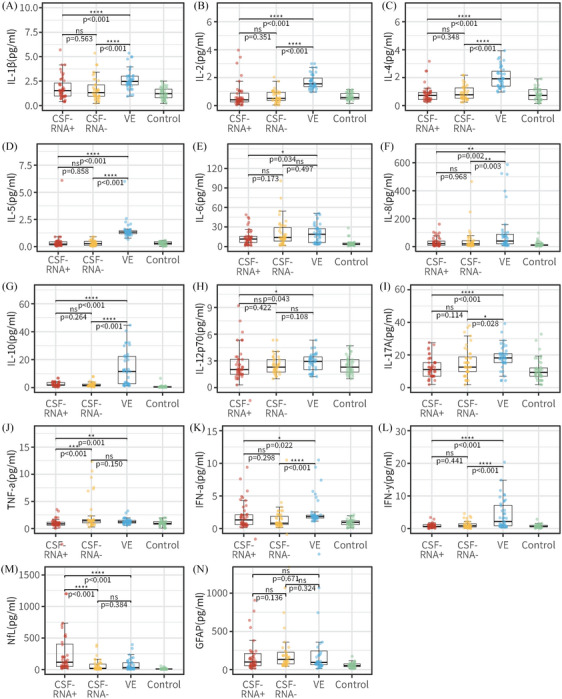
Comparison of the SARS‐CoV‐2 related parameters and laboratory parameters in Neuro‐COVID patients, patients with viral encephalitis and controls. CSF Il‐1β (A), IL‐2 (B), IL‐4 (C), IL‐5 (D), IL‐6 (E), IL‐8 (F), IL‐10 (G), IL‐12p70 (H), IL‐17A (I), TNF‐α (J), IFN‐α (K), and IFN‐γ (L) determined by immunofluorescence technology. Plasma NfL (M) and GFAP (N) from SiMoA Platform analysis. Comparisons between RNA^CSF^‐positive group (red), RNA^CSF^‐negative group (yellow), viral encephalitis group (blue) and control (green) done using the Mann–Whitney *U* test. *p < 0.05; **p < 0.01, ***p < 0.001, ****p < 0.0001. CSF, cerebrospinal fluid; IL, interleukin; TNF, tumor necrosis factor; IFN, interferon; NfL, neurofilament light chain; GFAP, glial fibrillary acidic protein.

In contrast with RNA^CSF^‐positive groups, the CSF total protein concentration (median [IQR], 0.37 [0.27–0.61] vs 0.46 [0.29–1.22] g/L; *p *= 0.012) was found to be significantly higher in RNA^CSF^‐negative group, and OCB results in positive rates were significantly increased in comparison with the other group (20.16 vs. 8.14%, *p *= 0.027). Therefore, it seems the integrity of BBB in group RNA^CSF^‐positive group appears to be better maintained. On the other hand, we observed significantly elevated serum levels of NfL protein (median [IQR], 114.9 [48.59–422.7] vs. 17.94 [10.48–94.12] pg/L; *p <* 0.001) in the RNA^CSF^‐positive group compared with the negative group, with a notable and statistically significant difference. In comparison with patients with VE, the RNA^CSF^‐positive group displayed notably more substantial neuronal damage (median [IQR], 114.9 [48.59–422.7] vs. 31.87 [11.19–114.4] pg/L; *p* < 0.0001) (refer to Figure 4 and Table S2). In contrast, the levels of serum GFAP levels did not exhibit a significant difference between the two groups (Table 2). These may suggest that the SARS‐CoV‐2 upon CNS invasion can cause more direct and pronounced damage to neurons or nerve fiber.^17^, ^18^, ^35^

After infection with SARS‐CoV‐2, there can be a robust activation of the immune response, leading to a cytokine storm that mediates nonspecific immune damage to human organs or tissues.^10^ To comprehend the inflammatory changes within the CNS of Neuro‐COVID patients, we conducted a detailed analysis of cytokine levels in the CSF across various patient groups (refer to Figure 4 and Table S2). In the RNA^CSF^‐positive group, eleven CSF cytokines demonstrated significantly decreased levels compared with the VE group, including IL1β, IL‐2, IL‐4, IL‐5, IL‐6, IL‐8, IL‐10, IL‐12p70, IFN‐γ, TNF‐α, and IFN‐α (*p* < 0.05). Notably, CSF TNF‐α was markedly lower in the RNA^CSF^‐positive group than in the RNA^CSF^‐negative group (median [IQR], 0.87 [0.62–1.21] vs. 1.44 [0.87–1.64] pg/L; *p* < 0.001). Interestingly, the levels of CSF IL1β, IL‐2, IL‐4, IL‐5, IL‐6, IL‐8, IL‐10, IL‐12p70, IFN‐γ, or IFN‐α did not show significant alterations in the RNA^CSF^‐positive group compared with the RNA^CSF^‐negative group and control group. These cytokines, which were elevated in the VE group, displayed a decrease in the RNA^CSF^‐positive group, resembling levels seen in the control group. This observation may suggest that the immune response in the CNS of Neuro‐COVID is not as robust as anticipated.

### 2.5 Neuro‐specific antibodies are not commonly found in Neuro‐COVID

Viral infections in the CNS are significant risk factors or triggers for autoimmune encephalitis, and the detection of neuro‐specific autoantibodies is a crucial marker.^36^, ^37^, ^38^ Therefore, following events of VE or viral invasion of the CNS, it is essential to observe and confirm potential cases of autoimmune encephalitis. We conducted screening for neuro‐specific autoantibodies in the CSF sample of all Neuro‐COVID patients and identified only one case had N‐methyl‐d‐aspartate receptor (NMDAR) antibody (Patient 1) and one case had GFAP antibody positivity (Patient 2), respectively originating from RNA^CSF^‐positive and negative group.

Table 3 details the comparisons between the two patients who have also been identified with neuro‐specific autoantibodies. Patient 1 presented generalized epileptic seizures and psychiatric disturbances, then further testing for autoimmune encephalitis antibodies showed the presence of anti‐NMDAR antibodies in both CSF and serum, with titers of 1:100 and 1:10, respectively. Initially, he received antiviral treatment but produced only a marginal effect. When NMDAR antibodies were detected, treatment attempts with intravenous immunoglobulin (IVIg) (a dosage of 0.4 g/kg/day for 5 days) were undertaken and the immunotherapy response was good. Patient 2 presented headache, dizziness, and rapid cognitive impairment after COVID‐19 infection. CSF analysis identified the CNS demyelination disease antibodies with GFAP‐IgG (titer of 1:1 in CSF). Following antiviral and IVIg therapy, the patient's headache disappeared and cognitive function showed some improvement.

**TABLE 3:** Clinical, serologic, and CSF characteristics of the Neuro‐COVID patients with autoimmune antibodies.

	Patient 1	Patient 2
Demographic characteristics		
Age at onset, years	M	M
Sex, *n* (%), male	49	39
Baseline mRS	3	3
Total disease duration, days	9	12
Time from confirmed COVID‐19 to the onset, days	7	8
Sign and symptoms		
Headache	+	+++
Dizziness	+	++
Seizures	+++	–
Psychosis	+++	–
Cognitive decline	–	+++
Disturbance of consciousness	++	–
CSF		
Lymphocytosis	+	+
Intrathecal IgG synthesis^a^	+	+
SARS‐CoV RNA	+	–
Autoimmune antibody		
Antibody	NMDAR	GFAP
Titers (corresponding samples) ^b^	S 1:10; CSF 1:100	CSF 1:1
Treatment/ response		
Antiviral treatment	Poor	Good
Immunotherapies		
IVIg	Good	Good
IVMP	ND	ND

Symbols: − = none; + = mild; ++ = moderate; +++ = prominent.

Abbreviations: GFAP, glial fibrillary acidic protein; IVIg, IV immunoglobulin; IVMP, intravenous high dose methylprednisolone; ND, not done; NMDAR, N‐methyl‐d‐aspartate receptor; S, serum.

^a^ Including oligoclonal bands.

^b^ Before immunotherapy.

In summary, evaluating the clinical features, imaging changes, and medical history of both patients and referring to the diagnostic criteria proposed by Gruas and Dalmau for autoimmune encephalitis,^39^, ^40^, ^41^ it is highly probable that Patient 1 is diagnosed with anti‐NMDAR autoimmune encephalitis. On the other hand, Patient 2 does not meet the criteria for autoimmune encephalitis. The neuro‐specific autoantibodies detected in Patient 2′s CSF are more likely to be reactive antibodies resulting from SARS‐CoV‐2 infection or secondary to an inflammatory state, rather than pathogenic antibodies.

## 3 DISCUSSION

Numerous studies have documented the presence of SARS‐CoV‐2 RNA in blood, which suggests the possibility of viremia.^20^, ^42^, ^43^, ^44^ However, the reliability of positive results obtained from some autopsy brain tissue samples may be questionable due to the distribution of blood throughout different tissues.^45^, ^46^, ^47^, ^48^ Therefore, caution is warranted when interpreting evidence regarding the virus's potential to invade the nervous system.^49^ Given the complex and anatomically structured nature of the human brain, it is crucial to explore the evidence of infectious virions in the CSF.^50^ Unfortunately, limited studies have shown that the current viral presence in the CSF lacks sufficient viral RNA or culture evidence.^22^ In other words, sporadic‐positive results have been reported only through reverse‐transcription polymerase chain reaction (RT‐qPCR) and antigen detection methods.^51^ Consequently, it is prudent to interpret these findings with caution and to continue investigating the virus's neuroinvasiveness with a more thorough and detailed approach.^52^ In conclusion, despite the evidence indicating the presence of SARS‐CoV‐2 RNA in blood, the current evidence regarding the virus's presence in the CSF is limited and requires further exploration.^19^

Our research has yielded significant and groundbreaking findings regarding the enhanced invasion of SARS‐CoV‐2 in the CNS. Specifically, we have utilized advanced molecular techniques, including RT‐qPCR and WGS, to confirm the presence of intact virus particles in the CSF.^53^, ^54^ This is the first study to report the detection of intact virus particles in the CSF, providing crucial and novel evidence of SARS‐CoV‐2′s potential to invade the CNS. Although the positive rate is not of epidemiological significance, we have also identified that the neurotoxicity resulting from the virus's neurotropic invasion may be time dependent. By analyzing the clinical characteristics of RNA^CSF^‐positive patients, we found that SARS‐CoV‐2 is more likely to affect the cerebral cortex or induce encephalitis‐associated neurological syndromes, resulting in rapid disease progression with typical psychiatric symptoms and prominent imaging. This finding is significantly different from previous studies examining the incidence of neurological manifestations and long‐term neurological outcomes, indicating that the pathogenesis of acute neurological syndromes associated with SARS‐CoV‐2 is diverse.^55^, ^56^, ^57^, ^58^ Additionally, our results suggest that SARS‐CoV‐2 infection may be an indirect cause of systemic inflammation processes and CNS immune activation, as we observed a high incidence of cerebellum and spinal cord injury conditions in RNA^CSF^‐negative patients. In summary, our research has provided significant and novel evidence regarding the potential of SARS‐CoV‐2 to invade the CNS and the diverse pathogenesis of acute neurological syndromes associated with the virus.^59^, ^60^, ^61^, ^62^ Further research is necessary to better understand the mechanisms underlying SARS‐CoV‐2′s neurotropic invasion and its potential long‐term effects on the CNS.^63^ Although our research has identified significant differences in clinical manifestations between RNA^CSF^‐positive and negative patients, we found no substantial differences in treatment protocols, length of hospital stays, or short‐term prognosis. This suggests that current treatment options are effective in managing the symptoms of CNS invasion by SARS‐CoV‐2. However, it is important to note that the absence of damage at present does not guarantee a lack of future consequences. As the virus continues to evolve and acquire new neurotropic or neuroinvasive properties, it may inflict collateral damage to the brain.^4^, ^64^, ^65^ Therefore, further studies are needed to investigate the long‐term effects of SARS‐CoV‐2 invasion of the CNS. In light of these findings, therapeutic strategies should focus on maximizing coverage of different neuropathogenesis mechanisms after SARS‐CoV‐2 infection, to comprehensively restrain both the direct viral invasion and the indirect immune‐mediated response. This approach could potentially prevent or mitigate long‐term neurological damage caused by SARS‐CoV‐2.

On the other hand, the laboratory findings in the CSF of Neuro‐COVID patients offer valuable insights into the neurological impact of SARS‐CoV‐2. In the study, we include a comparative analysis with previously diagnosed VE patients and a control group, aiming to assess neurologic damage and BBB integrity in Neuro‐COVID cases. In the RNA^CSF^‐negative group, a significantly higher concentration of CSF total protein and an increased rate of positive OCBs indicate potential differences in BBB integrity compared with the RNA^CSF^‐positive group. This suggests that the BBB in the RNA^CSF^‐positive group might be better maintained, potentially acting as a protective factor against neurologic damage.^49^, ^66^, ^67^ However, elevated serum levels of NfL protein in the RNA^CSF^‐positive group, even compared with patients with VE, imply severe neuronal damage upon SARS‐CoV‐2 invasion of the CNS. In contrast, levels of serum GFAP did not show a significant difference between the two groups, suggesting that the direct and pronounced damage caused by SARS‐CoV‐2 may be more specific to neurons or nerve fibers.^17^, ^18^, ^65^, ^68^, ^69^, ^70^ Furthermore, the study delves into the immune response by analyzing cytokine levels in the CSF of various patient groups. Interestingly, Neuro‐COVID patients exhibited generally lower CSF cytokine levels compared with those with VE, and levels were similar to the control group. The lower level of TNF‐α in the RNA^CSF^‐positive group compared with the negative group hints at a less intense immune response in the CNS of Neuro‐COVID patients than expected.^71^, ^72^, ^73^ In scientific and medical terms, these results contribute to our understanding of the complex interplay between SARS‐CoV‐2 and the nervous system. The maintenance of BBB integrity in the RNA^CSF^‐positive group may indicate a protective mechanism, but the heightened neuronal damage suggests a direct impact of the virus on the CNS. The observed immune response, while robust, appears to be less intense than anticipated, highlighting the unique nature of the neuroinvasion by SARS‐CoV‐2. These findings have significant implications for both the scientific community and clinicians in comprehending the neurological manifestations of COVID‐19 and guiding potential therapeutic interventions.

Furthermore, the detection of neuro‐specific autoantibodies in Neuro‐COVID patients holds significant scientific and medical value, providing crucial insights into the potential autoimmune responses triggered by SARS‐CoV‐2 infection in the CNS. Firstly, the identification of NMDA receptor (NMDAR) antibodies in Patient 1 and GFAP antibodies in Patient 2 underscores the link between SARS‐CoV‐2 infections, CNS invasion, and autoimmune encephalitis. These findings align with the established understanding that SARS‐CoV‐2 infections are substantial risk factors for autoimmune encephalitis, emphasizing the importance of monitoring and confirming potential cases post‐viral invasion. The detailed comparison in Table 3 between the two patients with neuro‐specific autoantibodies further enhances our understanding.^36^, ^37^, ^38^ Patient 1, diagnosed with anti‐NMDAR autoimmune encephalitis, presented with generalized epileptic seizures and psychiatric disturbances. The successful response to IVIg therapy suggests the therapeutic potential of targeted interventions in managing autoimmune encephalitis associated with SARS‐CoV‐2 infection. In contrast, Patient 2, who did not meet the criteria for autoimmune encephalitis, exhibited neuron‐specific autoantibodies likely to be reactive antibodies resulting from SARS‐CoV‐2 infection or an inflammatory state. This insight is pivotal in distinguishing between pathogenic and reactive antibodies, guiding appropriate therapeutic strategies. Evaluating these findings in the context of diagnostic criteria proposed by Gruas and Dalmau for autoimmune encephalitis adds credibility to the conclusions. The differential diagnosis and treatment response in Patients 1 and 2 illustrate the nuanced nature of autoimmune responses post‐SARS‐CoV‐2 infection, emphasizing the need for tailored approaches based on individual patient profiles.^39^, ^40^, ^41^ In summary, the detection of neuro‐specific autoantibodies in Neuro‐COVID patients not only contributes to our understanding of autoimmune responses triggered by SARS‐CoV‐2 but also provides a foundation for personalized treatment strategies. These findings underscore the intricate interplay between viral infections and autoimmune reactions in the CNS, offering valuable insights for clinicians and researchers working towards effective interventions and management protocols for neurological complications associated with COVID‐19.

Our study has several limitations. First of all, patients in this study occurred at an epoch during a surge period of the pandemic, the limited sample size as well as the short time window have restricted the interpretation and generalization of the current finding. For the restriction of RNA enrichment and RT‐qPCR technology sensitivity, the RNA^CSF^‐negative patients might have been in their initial infection phase or have lower viral load so they may have been misclassified. Second, this study was limited to determining the evidence of the virus's direct invasion in CNS by CSF testing, therefore patients with the neurological syndrome who did not undergo lumbar puncture were not included. This may underestimate the true number of neurological syndromes associated with SARS‐CoV‐2. Third, because of the research urgency and restrictions of laboratory biosafety, we did not culture CSF samples, and hence so could not obtain relevant information on the replication, proliferation, and biological activity of the SARS‐CoV‐2 in the CNS.^74^ Therefore, a prospective study including a larger sample size will be needed to help establish whether SARS‐CoV‐2 RNA detected in CSF is causal or coincidental in such patients.

In summary, this multicenter study has unveiled the presence of viral RNA and antigens in the CSF of individuals diagnosed with SARS‐CoV‐2 infection and acute neurological syndrome. Notably, patients with viral RNA in their CSF displayed more pronounced symptoms of acute brain disease. Addressing COVID‐19, both acute and post‐acute neuropsychiatric consequences, particularly cognitive deficits colloquially referred to as “COVID fog,” are raising public concern. This includes issues with memory, attention, language fluency, and daily problem‐solving persisting for months after infection. Brain fog is a complex phenomenon in clinical practice, involving both objective and subjective factors. A systematic review of 81 studies found that^75^ 20–30% of individuals with COVID‐19 experienced persistent cognitive impairment 12 or more weeks after diagnosis. While the cause remains unclear, whether related to hypoxia, inflammatory factors, or direct viral effects, it warrants further attention.^76^ In our study, RNA^CSF^‐positive patients demonstrated more acute encephalopathy manifestations, such as seizures and psychosis. These findings underscore the increasing invasion or affinity of SARS‐CoV‐2 for the CNS, emphasizing the need for clinicians to explore objective evidence across various dimensions for personalized treatment strategies.

## 4 MATERIALS AND METHODS

### 4.1 Study design and patients

This study was performed from December 16, 2022 to January 15, 2023. This multicenter, cross‐sectional study included hospitalized patients who were confirmed with SARS‐CoV‐2 infection during the Omicron Wave by RT‐qPCR‐positive assay testing on the nasopharyngeal specimens. They were admitted to West China Hospital of Sichuan University, Chengdu Shang Jin Nan Fu Hospital, and West China Tianfu Hospital and had also undergone a diagnostic lumbar puncture because of neurologic symptoms or as part of a research protocol (Figure 1). For patients with multiple CSF examinations, only the first reported data were included for analysis. The study protocol was approved by The West China Hospital Ethics Committee (2023 [30]). All the participants (or next of kin if the patient was not able) provided written informed consent. All the data analyzed in the study were strictly anonymous. The report follows the Strengthening the Reporting of Observational Studies in Epidemiology (STROBE) reporting guideline for cross‐sectional studies.

The case definition for Neuro‐COVID included any person with confirmed SARS‐CoV‐2 infection admitted to the hospital with the symptoms lasting ≥24 h and the presence of the following “Neuro‐COVID” criteria: (1) signs and symptoms commonly associated with COVID‐19 including headache, vertigo, and sleep disorder; (2) new onset neurological signs by clinical diagnoses after SARS‐CoV‐2 infection (including psychosis, seizure and/or status epilepticus, limb weakness, blurred vision, disturbance of sensation, cognitive function declined rapidly, disturbance of consciousness, dyskinesias, and movement disorders); and radiographic imaging evidence of abnormalities consistent with neurological signs. Exclusion criteria were patients with malignant tumors, craniocerebral trauma, definite intracranial infection with other pathogens, and severe preexisting neurological dysfunction. Fever was not considered a supportive feature for CNS infection (as indicated by the standard criteria), as it is highly prevalent in COVID‐19.

### 4.2 Clinical information and sample collection

Clinical data including demographic data, clinical phenotype, past medical history, in‐hospital stay, treatment modalities, brain MRI, lung CT/chest X‐ray, and CSF examination were collected from medical records and electronic databases. Preexisting neurological disorders included cerebrovascular disease, epilepsy, myelopathy, Parkinson's disease, dementia, cognitive disorder, and unspecified neurological disorders. Evaluation of clinical functional outcome was assessed through the mRS evaluation made by the three experienced neurologists both on admission and at discharge from the hospital. Recovery was defined as a decrease in the mRS score ≥1 point at discharge compared with admission. Poor response to treatment was defined as no improvement in the mRS score since admission.

Lumbar puncture was performed within 3 days of hospitalization and before treatment. Patient samples, including EDTA anticoagulated plasma and nonanticoagulated serum, are simultaneously collected, processed through standard centrifugation, and separation procedures, and promptly stored at low temperatures. All the samples were processed within an hour to ensure optimal quality. All operations follow standard operating procedures developed by West China Hospital of Sichuan University (*SOP.WCH‐LM‐HEM‐B*).

### 4.3 Total DNA/RNA extraction in CSF sample and RT‐qPCR assay

First, every 10 μL protease K solution (10 mg/mL) and 10 μL carrier RNA (1 mg/mL) were added to a sample tube (1.5 mL). Second, 1000 μL of each CSF sample (sterile polyethylene tube storage) was added to the sample tube. Then, the total RNA of all CSF samples was extracted using a Concert viral RNA kit (RC1016: Concert Biotech, Xiamen, China) with an HF16 nucleic acid purification instrument according to the manufacturer's guidelines. RC1016(210) extraction kit with HF Automated Nucleic Acid Extractor provides 1000 μL sample nucleic acid purification solution therefore, there are more opportunities to purify nucleic acids in micro concentration virus sample.

Real‐time PCR (RT‐PCR) was performed by amplifying two target genes, open reading frame 1ab (ORF1ab) and nucleocapsid protein (N), using a qRT‐PCR kit (Sansure Biotech Inc., Changsha, China) with a RT‐PCR thermal cycler (ABI 7500 system; Applied Biosystems instruments, Waltham, MA, USA). Each test needs to be repeated, and the results are consistent twice before they are accepted and recorded. The primers used were as follows:

SARS‐CoV‐2_ORF1ab‐F: 5′‐CCCTGTGGGTTTTACACTTAA‐3′,

SARS‐CoV‐2_ORF1ab‐R: 5′‐ACGATTGTGCATCAGCTGA‐3′,

SARS‐CoV‐2_ORF1ab‐P: 5′‐FAM‐CCGTCTGCGGTATGTGGAAAGGTTATGG‐BHQ1‐3′,

SARS‐CoV‐2_N‐F: 5′‐GGGGAACTTCTCCTGCTAGAAT‐3′,

SARS‐CoV‐2_N‐R: 5′‐CAGACATTTTGCTCTCAAGCTG‐3′, and

SARS‐CoV‐2_N‐P: 5′‐FAM‐TTGCTGCTGCTTGACAGATT‐TAMRA‐3′.

When ORF1ab and N genes were both positive (cycle threshold [Ct] < 37), the results were considered certain positive for SARS‐CoV‐2. When the results showed no Ct value or Ct ≥ 40 at the two detection sites, the results were considered negative for SARS‐CoV‐2.

### 4.4 SARS‐CoV‐2 whole viral sequencing (WGS), mapping, and phylogenetic analysis

Given the stringent requirements for WGS analysis targeting viral genomic sequences, a meticulous selection of viral genome templates is imperative. To ensure the integrity of WGS analysis outcomes and enhance the reliability of evolutionary tree construction and traceability analyses, we exclusively opt for samples that exhibit positive results through RT‐PCR detection with a Ct < 25 for WGS analysis.

Total RNA was extracted from the clinical specimens by Magnetic Viral DNA/RNA kit (Genskey, China). Sequencing libraries were constructed with the ATOPlex SARS‐CoV‐2 Full Length Genome Panel V3.0 (MGI, China), following the manufacturer's instructions. The DNB was generated by DNBSEQ OneStep DNB Make Reagent Kit (MGI) and sequenced on a MGIseq‐2000 instrument at Genskey China with a single‐end 100 sequencing set (MGI). The sequencing data were first quality‐controlled and then mapped to the first Wuhan SARS‐CoV‐2 genome (MN908947.3). The SARS‐CoV‐2 clade was analyzed by Nextclade (v2.9.1). The phylogenetic tree was constructed using RAxML, iTOL, and consensus genomes from each sample.

### 4.5 Metatranscriptomic sequencing

The quality and concentration of DNA samples were monitored by a Qubit fluorometer (Thermo Fisher Scientific, MA, USA), and metagenomics libraries were constructed by a QIAseq Ultralow input library kit (Genskey 1906, Beijing, China). Library quality control was performed with a Qubit fluorometer (Thermo Fisher Scientific) and Agilent 2100 Bioanalyzer (Agilent Technologies, Palo Alto, CA, USA). The qualified libraries with different barcode labeling were pooled and sequenced on an Illumina Nextseq 550 platform (Illumina, San Diego, CA, USA). In parallel with the clinical samples, positive controls and negative controls (including a nontemplate control [NTC]) were also set for mNGS detection with the same procedure and bioinformatics analysis. The NTC samples enabled estimation of the number of background reads except for each taxon. High‐quality data were generated after filtering out adapter, low‐quality, low‐complexity, and shorter reads. Next, human reads were removed by mapping reads to the human reference genome (GRCh38) using Bowtie2. The remaining data were aligned to the microbial genome database(https://ftp.ncbi.nlm.nih.gov/genomes/) using Burrows‐Wheeler alignment. The read number and RPM of each detected pathogen were calculated, and the microbial composition was determined. The formula for calculating RPM was as follows: RPM of pathogen = (number of reads mapped to the pathogen × 106)/(total number of mapped reads from given library).

### 4.6 SARS‐CoV‐2 antibody detection

Chemiluminescence immunoassays and lateral flow immunoassays for anti‐SARS‐CoV‐2 antibodies were tested by Wantai Bio, including IgM, IgG, and neutralizing antibodies. This was a two‐step immunoassay with a chemiluminescent microparticle technology principle. Microparticles coated with SARS‐CoV‐2 antigen were combined with assay diluent followed by incubation. The antibodies present in the participant's serum bind with the antigen‐coated microparticle. An anti‐human target antibody labeled with acridinium conjugate was added followed by pretrigger and trigger solution. The test reaction was measured by system optics and expressed as a relative light unit (RLU). The level of RLU was directly proportional to the amount of target antibody. It was then compared with the calibrator RLU to determine the presence and absence of target antibodies against SARS‐CoV‐2. The CSF samples were run undiluted (50 μL per well). The detected RLU value will be converted into a qualitative test result.

### 4.7 SARS‐CoV‐2 antigen detection

Detection of SARS‐CoV‐2 nucleocapsid and spike proteins was performed using MSD SPLEX CoV‐2 N and MSD S‐PLEX CoV‐2 S assay kits (Meso Scale Discovery). The MSD S‐PLEX assay kits (Meso Scale Discovery, Rockville, MD) employ a sandwich immunoassay format and electrochemiluminescence (ECL) detection. The assays are carried out in specially designed 96‐well plate consumables having integrated screen‐printed carbon ink electrodes on the bottom of each well that are used as solid‐phase supports for binding reactions and as the source of electrical energy for inducing ECL from ECL labels in binding complexes on their surfaces. The kits use MSD's ultra‐sensitive S‐PLEX ECL format, which provides additional signal enhancement and sensitivity relative to conventional ECL formats. Sample quantitation was achieved using a calibration curve generated with a recombinant antigen standard, and fit a four‐parameter logistic (4PL) model. The LOD for all S‐PLEX assays was determined as the concentration (based on a 4PL fit to a calibration curve) that provides a signal 2.5 standard deviations above the blank signal.

The N antigen assay utilizes recombinant full‐length N‐protein as standard and monoclonal capture/detection antibodies generated by MSD against the full‐length recombinant N protein. The S antigen assay utilizes the recombinant receptor binding domain (RBD) of the S1 subunit of the spike protein as standard and monoclonal capture/detection antibodies generated by MSD against recombinant RBD. The assay cut‐offs for classifying samples as N or S antigen‐negative or positive were established in previous clinical studies of respiratory or plasma samples.

### 4.8 Main laboratory detection

The CSF karyocyte count was determined using an optical microscope after inhaling 200 microliters of CSF sample into a cell centrifuge at a low speed of 1000 rpm/3 min (centrifugal radius: 13 cm). Total protein and intrathecal immunoglobulin concentrations were evaluated through nephelometry. OCBs were detected using isoelectric focusing, and silver nitrate staining was employed to assess the integrity of the blood–CSF barrier.

The CSF sample cytokine detection utilizes immunofluorescence technology with a reagent kit containing 12 capture microspheres coated with specific antibodies for L‐1β, IL‐2, IL‐4, IL‐5, IL‐6, IL‐8, IL‐10, IL‐12p70, IFN‐γ, TNF‐α, and IFN‐α. These microspheres selectively bind to corresponding cytokines in the test sample, forming a sandwich complex (capture microsphere + test sample + PE‐labeled detection antibody). The fluorescence intensity of this complex is analyzed to determine their levels, aiding in immune function assessment. Detection ranges are 2.5–2500 pg/mL for IL‐1β, IL‐2, IL‐4, IL‐5, IL‐6, IL‐8, IL‐10, IL‐12p70, IFN‐γ, TNF‐α, and IFN‐α, and 10–2500 pg/mL for IL‐17A. Results within range are directly reported; if exceeding 2500 pg/mL, a 1:4 dilution is recommended. Values below 2.5 pg/mL for IL‐1β, IL‐2, IL‐4, IL‐5, IL‐6, IL‐8, IL‐10, IL‐12p70, IFN‐γ, TNF‐α, and IFN‐α, and <10 pg/mL for IL‐17A are reported as below the detection limit.

The Neuro Plex Kit from Quanterix Corporation (Lexington, MA, USA) was utilized for analyzing NfL and GFAP levels in plasma samples, employing a bead‐based ultrasensitive technique on the SR‐X instrument of the SiMoA Platform. Following the manufacturer's instructions, the protocol involved calibration standard curves for quantification and analog and digital controls to set detection limits. Duplicate applications of higher and lower controls, along with all samples, were performed at a 1:4 dilution. In a 96‐well plate, paramagnetic carboxylated microspheres and detector buffer were added, followed by a 30‐min incubation at 35°C and 800 rpm. After washing, Streptavidin β‐galactosidase (SβG) was added, and the plate underwent another incubation for 10 min at 35°C and 800 rpm. Following a second wash, the plate was incubated for an additional minute under the same conditions, dried, and inserted into the SR‐X equipment. Quanterix software conducted and recorded the analysis, incorporating dilution correction. Reference values for each biomarker were established at 0−500 pg/mL for NfL and 0−1000 pg/mL for GFAP. The lower detection limits for NfL and GFAP were 0.136 and 0.276 pg/mL, respectively, with ranges of 2000 pg/mL for NfL and 40,000 pg/mL for GFAP. It is essential to note that plasma samples were diluted 1:4 for analysis, and the SiMoA software considers this for the final concentration calculation.

### 4.9 Neuro‐specific autoantibody detection

To confirm and rule out the potential presence of neuro‐specific autoantibodies, antibody testing was conducted on CSF samples from all patients in our cohort. In particular, patients’ CSF sample were used transfection cell‐based indirect immunofluorescence tested for autoimmune encephalitis panel (anti‐NMDAR/LGI1/CASPR2/GABAAR/GABABR/AMPA1/AMPA2/DPPX/lgLON5/mGluR5/GlyRα1 antibodies) and paraneoplastic panel(anti‐Hu/Yo/GAD65/Titin/Recoverin/PKCγ/Zic4/Tr(DNER)/SOX1/Ma1/Ma2/Amphiphysin/CV2/Ri antibodies) by MYBiotech Co. Ltd., Shaanxi, China.

### 4.10 Statistical analyses

The data has been expressed as medians with IQRs (25–75%). The differences between the continuous variables were analyzed by the Mann–Whitney *U* test. The various categorical variables were compared using two tests, two analyses with continuity correction, or Fisher's exact test, as appropriate. A two‐sided *p* value of less than 0.05 was considered statistically significant. All the statistical analyses were conducted using SPSS version 25.0 for Mac. Figures were generated with GraphPad Prism 8 and R version 3.5.0 for Mac.

## AUTHOR CONTRIBUTIONS

M. W. and J. L. had full access to all of the data in the study and take responsibility for the integrity of the data and accuracy of the data analysis. J. W. and B. Y. contributed equally to the manuscript. M. W., J. L., J. W., and Y. R. conceptualized and designed the study. M. W., J. W., Y. R., L. L., Y. Y., S. X., and M. T. contributed with the acquisition, analysis, or interpretation of data. M. W., J. W., L. L., and W. X. drafted the manuscript. W. L., L. C., D. Z., B. Y., and J. L. performed critical revision of the manuscript for important intellectual content. M. W. and J. W. performed the statistical analysis. L. L., S. X., Y. X., W. L., L. C., D. Z., B. Y., and J. L. provided administrative, technical, or material support. All authors have read and approved the final manuscript.

## CONFLICT OF INTEREST STATEMENT

The authors declare no conflict of interest.

## ETHICS STATEMENT

The West China Hospital Ethics Committee (2023 [30]) approved the study. All participants provided written consent.

## Supporting information

Supporting information

## Data Availability

The data supporting the findings of this study are available within the article and from the corresponding author upon reasonable request.
