# Supplementary material for: Current clinical findings of acute neurological syndromes after SARS‐CoV‐2 infection
Source: MedComm (2020). 2024 Mar 9;5(3):e508. doi: 10.1002/mco2.508 (PMC10924641; doi:10.1002/mco2.508)
Supplement: Supplementary file 1 — Supporting information [file MCO2-5-e508-s001.docx]

**Current clinical findings of acute neurological syndromes after SARS-CoV-2 infection**

#Minjin Wang^1,2,3^, MD, Ph.D; #Jierui Wang^1,3^, MD; Yan Ren^2^, Ph.D; Lu Lu^1,3^, MD; Weixi Xiong^1,3^, MD;

Lifeng Li^4^, Ph.D; Songtao Xu^5^, Ph.D; Meng Tang^2^, Ph.D; Yushang Yuan^2^, Ph.D; Yi Xie^2^, Ph.D; Weimin Li^6^, MD;

Lei Chen^1,3^, MD; *Dong Zhou^1,3^, MD; *Binwu Ying^2^, Ph.D; *Jinmei Li^1,3^, MD, Ph.D.

1. Department of Neurology, West China Hospital of Sichuan University, China;
2. Department of Laboratory Medicine, West China Hospital of Sichuan University, China；
3. Institute of Brain Science and Brain-inspired Technology of West China Hospital, Sichuan University, China；
4. Genskey Medical Technology Co., Ltd, Beijing, China;
5. State Key Laboratory for Infectious Disease Prevention and Control, National Institute for Viral Disease Control and Prevention, Chinese Center for Disease Control and Prevention, Beijing, China;
6. Department of Respiratory and Critical Care Medicine, West China Hospital, Sichuan University, China;

**Corresponding author:**

Jinmei Li, MD, Ph.D.

1. mail: lijinmei@wchscu.cn.

Binwu Ying, Ph.D.

E-mail: yingbinwu@scu.edu.cn.

Dong Zhou, MD, Ph.D.

1. mail: zhoudong66@yahoo.de

# and *: These authors have contributed equally to the manuscript.

Number of words in the manuscript: 6163

Number of words in the abstract: 190

Number of characters in the title: 10

Number of references: 76

Number of tables: 3

Number of figures: 4

**Figure legend**

Figure S1 | Phylogeny and genome statistics of 10 SARS-CoV-2 isolates. 9 CSF samples and 1 matched NPS sample showed high coverage of the complete SARS-CoV-2 genome (coverage 10X≥90% and coverage 100X≥60% against the first Wuhan SARS-CoV-2 genome)

A.The phylogenetic tree was constructed using RAxML, iTOL and the consensus genomes from each analyzed sample.

B.The yellow square indicates the different sample types whereas the blue square represents different Pango lineage detected by Nextclade. Three bubble plots illustrating the sequencing coverage (1X, 10X, and 100X) for each sample against the first Wuhan SARS-CoV-2 genome.

C.Mapping and semi-log depth of coverage of the sequencing reads for each sample against the first Wuhan SARS-CoV-2 genome.

Table S1 Whole genome sequencing and genotyping of SARS-COV-2 infected samples

| PatientID | SampleID | Sample Type | No. of RawReads | No.of SARS-COV-2_Reads | Avg depth of SARS-COV-2 | Coverage 1x of SARS-COV-2(%) | Coverage 10x of SARS-COV-2(%) | Coverage 100x of SARS-COV-2(%) | clade | Nextclade_pango |
| --- | --- | --- | --- | --- | --- | --- | --- | --- | --- | --- |
| 1359 | NPS1359 | Nasopharyngeal swab | 16819924 | 880735 | 2790.38 | 100 | 100 | 99.33 | 22B (Omicron) | BA.5.2.36 |
| 1324 | NPS1324 | Nasopharyngeal swab | 8867493 | 2179587 | 7123.72 | 100 | 100 | 100 | 22B (Omicron) | BA.5.2.36 |
| 1310 | NPS1310 | Nasopharyngeal swab | 8189816 | 2778671 | 9063.38 | 100 | 100 | 100 | 22B (Omicron) | BA.5.2.36 |
| 1340 | NPS1340 | Nasopharyngeal swab | 6034140 | 2737922 | 8927.83 | 100 | 100 | 100 | 22B (Omicron) | BA.5.2.36 |
| 1329 | NPS1329 | Nasopharyngeal swab | 3693862 | 144919 | 439.01 | 100 | 99.7 | 77.16 | 22B (Omicron) | BA.5.2.36 |
| 1356 | NPS1356 | Nasopharyngeal swab | 3470587 | 1464071 | 4767.64 | 100 | 100 | 100 | 22B (Omicron) | BA.5.2.36 |
| 1325 | NPS1325 | Nasopharyngeal swab | 3042392 | 664768 | 2138.76 | 100 | 100 | 99.11 | 22B (Omicron) | BA.5.2.36 |
| 1326 | NPS1326 | Nasopharyngeal swab | 3036306 | 214662 | 679.68 | 99.69 | 99.21 | 89.83 | 22B (Omicron) | BA.5.2.36 |
| 1305 | NPS1305 | Nasopharyngeal swab | 2867314 | 119500 | 360.13 | 97.6 | 87.98 | 63.66 | 22B (Omicron) | BA.5.2.36 |
| 1346 | NPS1346 | Nasopharyngeal swab | 2771914 | 78359 | 222.46 | 96.8 | 82 | 47.42 | 22B (Omicron) | BF.7 |
| 1327 | NPS1327 | Nasopharyngeal swab | 2011944 | 16939 | 33.88 | 83.93 | 39.67 | 6.74 | 20B | B.1.1.161 |
| 1339 | NPS1339 | Nasopharyngeal swab | 373833 | 29742 | 92.14 | 99.64 | 87.67 | 23.62 | 22B (Omicron) | BA.5.2.36 |
| 472 | C472 | Cerebrospinal fluid | 8849941 | 14474 | 17.76 | 84.63 | 5.05 | 0.12 | 20A | B.1 |
| 6142 | C6142 | Cerebrospinal fluid | 8018568 | 2612270 | 8519.26 | 100 | 100 | 100 | 22B (Omicron) | BA.5.2.36 |
| 1324 | C1324 | Cerebrospinal fluid | 7446988 | 1805728 | 5894.94 | 100 | 100 | 100 | 22B (Omicron) | BA.5.2.36 |
| 6110 | C6110 | Cerebrospinal fluid | 6169322 | 12060 | 17.65 | 93.47 | 16.13 | 0.29 | 22B (Omicron) | BA.5.2.36 |
| 6690 | C6690 | Cerebrospinal fluid | 4869016 | 2846086 | 9337.88 | 100 | 100 | 100 | 22B (Omicron) | BA.5.2.36 |
| 6145 | C6145 | Cerebrospinal fluid | 4612080 | 500001 | 1603 | 99.81 | 97 | 90.26 | 22B (Omicron) | BA.5.2 |
| 1316 | C1316 | Cerebrospinal fluid | 4554660 | 34009 | 79.17 | 94.28 | 53.06 | 14.35 | 22B (Omicron) | BA.5.2.36 |
| 6001 | C6001 | Cerebrospinal fluid | 4144398 | 47763 | 97.22 | 87.83 | 21.2 | 13.51 | 20B | B.1.533 |
| 415 | C415 | Cerebrospinal fluid | 3938504 | 52299 | 134.76 | 93.78 | 55.4 | 28.56 | 22B (Omicron) | BA.5.2 |
| 6129 | C6129 | Cerebrospinal fluid | 3716441 | 18685 | 35.28 | 92.14 | 33.28 | 5.82 | 22B (Omicron) | BA.5.2 |
| 424 | C424 | Cerebrospinal fluid | 3404860 | 19543 | 37.58 | 85.91 | 38.82 | 8.52 | 22B (Omicron) | BA.5.2 |
| 396 | C396 | Cerebrospinal fluid | 3031006 | 59453 | 169.78 | 89.05 | 48.45 | 34.67 | 22B (Omicron) | BA.5.2.36 |
| 1346 | C1346 | Cerebrospinal fluid | 2867298 | 12052 | 15.85 | 75.19 | 2.57 | 0.72 | 20B | B.1.1.161 |
| 1329 | C1329 | Cerebrospinal fluid | 2807596 | 34044 | 86.93 | 86.86 | 44.89 | 19.88 | 22B (Omicron) | BA.5.2.36 |
| 1327 | C1327 | Cerebrospinal fluid | 2684632 | 135702 | 411.38 | 95.34 | 80.31 | 55.78 | 22B (Omicron) | BA.5.2.21 |
| 365 | C365 | Cerebrospinal fluid | 2232384 | 608339 | 1974.05 | 99.51 | 99.06 | 94.6 | 22B (Omicron) | BA.5.2.36 |
| 400 | C400 | Cerebrospinal fluid | 2190577 | 29795 | 84.22 | 75.55 | 18.97 | 15.67 | 22B (Omicron) | BA.5.2 |
| 6002 | C6002 | Cerebrospinal fluid | 2169380 | 16837 | 28.29 | 87.3 | 39.39 | 2.64 | 22B (Omicron) | BA.5.2.1 |
| 6147 | C6147 | Cerebrospinal fluid | 1923422 | 129935 | 405.67 | 95.77 | 84.12 | 62.34 | 22B (Omicron) | BA.5.2.36 |
| 6106 | C6106 | Cerebrospinal fluid | 1755291 | 29672 | 82.99 | 83.67 | 39.08 | 19.99 | 22B (Omicron) | BA.5.2 |
| 452 | C452 | Cerebrospinal fluid | 1597594 | 20426 | 48.98 | 73.07 | 25.47 | 12.29 | 20B | B.1.1 |
| 6059 | C6059 | Cerebrospinal fluid | 1561713 | 17229 | 42.98 | 80.94 | 43.33 | 7.79 | 22B (Omicron) | BA.5.2 |
| 1325 | C1325 | Cerebrospinal fluid | 1496835 | 39411 | 111.06 | 78.67 | 54.28 | 26.93 | 22B (Omicron) | BA.5.2 |
| 6289 | C6289 | Cerebrospinal fluid | 1378408 | 160187 | 518.28 | 98.18 | 93.38 | 73.93 | 22B (Omicron) | BA.5.2 |
| 6105 | C6105 | Cerebrospinal fluid | 1306119 | 200812 | 640.5 | 99.94 | 98.25 | 81.34 | 22B (Omicron) | BA.5.2.36 |
| 1359 | C1359 | Cerebrospinal fluid | 1142682 | 5817 | 6.99 | 53.36 | 0.19 | 0.12 | 20B | B.1.1 |
| 393 | C393 | Cerebrospinal fluid | 1097719 | 127162 | 408.86 | 96.57 | 90.93 | 66.8 | 22B (Omicron) | BA.5.2.36 |
| 1356 | C1356 | Cerebrospinal fluid | 1067377 | 5396 | 6.52 | 46.42 | 1.63 | 0.12 | 19A | B |
| 6148 | C6148 | Cerebrospinal fluid | 993231 | 14478 | 40.78 | 66.83 | 34.73 | 12.21 | 22B (Omicron) | BA.5.2 |
| 1326 | C1326 | Cerebrospinal fluid | 957737 | 36187 | 107.27 | 81.18 | 61.28 | 29.35 | 22B (Omicron) | BA.5.2 |
| 450 | C450 | Cerebrospinal fluid | 895058 | 396465 | 1274.5 | 100 | 100 | 98.8 | 22B (Omicron) | BF.7 |
| 1339 | C1339 | Cerebrospinal fluid | 832125 | 4245 | 5.09 | 44.04 | 0.34 | 0.12 | 19A | B |
| 1305 | C1305 | Cerebrospinal fluid | 824477 | 21432 | 59.01 | 78.11 | 52.87 | 14.78 | 22B (Omicron) | BA.5.2.36 |
| 478 | C478 | Cerebrospinal fluid | 766990 | 7452 | 11.38 | 41.36 | 7.93 | 1.23 | 19A | B |
| 406 | C406 | Cerebrospinal fluid | 545612 | 10167 | 27.02 | 52.01 | 24.89 | 7.99 | 20B | B.1.1.161 |
| 377 | C377 | Cerebrospinal fluid | 423706 | 4601 | 10.49 | 38.07 | 12.83 | 2.76 | 19A | B |
| 483 | C483 | Cerebrospinal fluid | 333422 | 3815 | 6.19 | 28.1 | 5.61 | 0.71 | 19A | B |
| 385 | C385 | Cerebrospinal fluid | 279467 | 4649 | 11.11 | 53.52 | 25.76 | 0.9 | 20B | B.1.1 |
| 404 | C404 | Cerebrospinal fluid | 259271 | 61562 | 199.5 | 94.16 | 88.28 | 55.03 | 22B (Omicron) | BA.5.2 |
| 1340 | C1340 | Cerebrospinal fluid | 174872 | 33147 | 106.93 | 100 | 97.75 | 32.68 | 22B (Omicron) | BA.5.2.36 |
| 401 | C401 | Cerebrospinal fluid | 114757 | 1026 | 1.2 | 11.1 | 0.12 | 0.12 | 19A | B |
| 380 | C380 | Cerebrospinal fluid | 68420 | 1683 | 4.45 | 30.89 | 11.34 | 0.32 | 19A | B |

**Supplemental Table. SARS-CoV-2 related parameters and biological characteristic of Neuro-COVID patients, patients with viral encephalitis and controls.**

|  | | | |  |
| --- | --- | --- | --- | --- |
| **Characteristic** | **RNA^CSF^ positive**  **n= 44** | **RNA^CSF^ negative**  **n= 40** | **VE**  **n= 40** | **Control**  **n= 40** |
| **CSF Cytokines** |  |  |  |  |
| Il-1β, median (IQR) (pg/ml) | 1.53 (0.96, 2.47) | 1.34 (0.95, 2.12) | 2.47 (2.08, 3.01) | 1.21 (0.8, 1.74) |
| IL-2, median (IQR) (pg/ml) | 0.41 (0.26, 0.93) | 0.51 (0.36, 0.95) | 1.55 (1.35, 2.1) | 0.56 (0.46, 0.85) |
| IL-4, median (IQR) (pg/ml) | 0.72 (0.43, 0.93) | 0.78 (0.522, 1.29) | 1.9 (1.37, 2.53) | 0.72 (0.42, 1.14) |
| IL-5, median (IQR) (pg/ml) | 0.23 (0.13, 0.45) | 0.27 (0.12, 0.49) | 1.34 (1.16, 1.47) | 0.3 (0.18, 0.44) |
| IL-6, median (IQR) (pg/ml) | 11.57 (5.89, 15.8) | 13.94 (7.89, 30.89) | 18.92 (5.89, 27.57) | 3.83 (2.72, 5.45) |
| IL-8, median (IQR) (pg/ml) | 20.04 (8.52, 38.93) | 18.77 (8.98, 42.9) | 38.83 (19.36, 93.51) | 9.81 (6.98, 14.9) |
| IL-10, median (IQR) (pg/ml) | 1.79 (1.52, 3.62) | 1.68 (1.04, 2.48) | 11.56 (2.72, 22.28) | 0.4 (0.31, 0.7) |
| IL-12p70, median (IQR) (pg/ml) | 2.03 (1.49, 3.16) | 2.3 (1.76, 3.16) | 2.93 (2.03, 3.49) | 2.3 (1.76, 3.16) |
| IL-17A, median (IQR) (pg/ml) | 11 (6.99, 15.29) | 12.51 (9.73, 20.09) | 18.16 (15.23, 20.83) | 9.36 (6.8, 12.37) |
| TNF-a, median (IQR) (pg/ml) | 0.87 (0.62, 1.21) | 1.44 (0.87, 1.64) | 1.23 (0.99, 1.47) | 0.95 (0.66, 1.29) |
| IFN-a, median (IQR) (pg/ml) | 1.32 (0.59, 2.16) | 0.82 (0.55, 2.03) | 1.82 (1.6, 2.06) | 0.95 (0.61, 1.25) |
| IFN-γ, median (IQR) (pg/ml) | 0.64 (0.44, 1.22) | 0.84 (0.45, 1.56) | 2.19 (0.86, 7.43) | 0.64 (0.44, 1.03) |
| **Plasma NfL, median (IQR) (pg/ml)** | 114.9 (48.59, 422.7) | 17.94 (10.48, 94.12) | 31.87 (11.19, 114.4) | 8.72 (5.53, 10.37) |
| **Plasma GFAP, median (IQR) (pg/ml)** | 98.05 (50.91, 220.8) | 133.1 (72.77, 236.6) | 94.3 (58.79, 276.7) | 51.01 (37.91, 75.46) |
| Abbreviations: CSF, cerebrospinal fluid; IL, interleukin; TNF, tumor necrosis factor; IFN, interferon; NfL, neurofilament light chain; GFAP, glial fibrillary acidic protein. | | | |  |

**Detailed instructions on RT-qPCR tests**

First, we enriched the total RNA in the cerebrospinal fluid in a modern and standard laboratory. The laboratory was thoroughly cleaned before and after each experiment to ensure a clean environment, and was strictly isolated from other types of SARS-CoV-2 clinical specimens, particularly respiratory samples. This was done to guarantee the accuracy of our results. Additionally, to further ensure the validity of our results, environmental samples from the lumbar puncture site and the laboratory work area were included in the RNA enrichment process as controls.

Next, we will conduct PCR testing on the RNA samples using two different RT-qPCR detection reagents. These two reagents have different primer sequences, allowing us to compare the results and eliminate potential laboratory PCR amplification product contamination. Only when the results from both RT-qPCR tests are consistent will we consider the results to be credible and record them for the experiment. Additionally, in every batch of PCR testing, we randomly insert environmental control samples, blank controls (pure water), and negative quality control samples to monitor all potential and latent laboratory contamination.

Finally, according to the instructions provided by the RT-qPCR assay kit, samples with a Ct value less than 40 are considered positive results. However, to mitigate potential random error and other non-specific PCR amplification effects, we only regard samples with a Ct value less than 35 as confirmed positive results and include them in our study to ensure accuracy in our result interpretation.

Through the aforementioned three steps, we have taken all necessary measures to eliminate potential risks of laboratory PCR contamination and ensure the accuracy and reliability of our results.

Furthermore, we would like to make a further statement regarding our innovation in the total RNA enrichment in the cerebrospinal fluid experiment, as well as the improvements made compared to previous studies. In our study, we require a minimum volume of 1 milliliter for each cerebrospinal fluid sample used for detection, although obtaining this amount can be challenging due to the precious nature of cerebrospinal fluid. In many previous studies, the volume used for detection was often 0.1 to 0.5 milliliters. Additionally, we have adopted an innovative approach of using specific magnetic bead separation and enrichment to enrich the total RNA in the cerebrospinal fluid, which has been shown to significantly improve the efficiency of RNA enrichment compared to traditional methods such as homogenization and filtration. We all know that enriching high-quality RNA samples from cerebrospinal fluid will significantly improve the sensitivity and accuracy of PCR test results. This also explains why we have been able to detect more SARS-CoV-2 virus RNA information from cerebrospinal fluid compared to other studies.
